# Co‐Opting MBNL‐Dependent Alternative Splicing Cassette Exons to Control Gene Therapy in Myotonic Dystrophy

**DOI:** 10.1002/ana.78024

**Published:** 2025-08-29

**Authors:** Samuel T. Carrell, Ellie M. Carrell, Ryan Giovenco, Beverly L. Davidson

**Affiliations:** ^1^ Department of Neurology University of Pennsylvania Philadelphia PA; ^2^ Raymond G. Perelman Center for Cellular and Molecular Therapeutics The Children's Hospital of Philadelphia Philadelphia PA; ^3^ Department of Pathology and Laboratory Medicine University of Pennsylvania Philadelphia PA

## Abstract

**Objective:**

Myotonic dystrophy type 1 (DM1) is a highly variable, multisystemic genetic disorder caused by a CTG repeat expansion in the 3′ untranslated region of *DMPK.* Toxicity is exerted by repeat‐containing *DMPK* transcripts that sequester muscleblind‐like (MBNL) proteins and lead to deleterious yet predictable changes in alternative splicing. To contend with high phenotypic and molecular variability that complicate application of viral‐based therapies, we develop and test a DM1‐responsive genetic element to control viral‐based therapeutic output.

**Methods:**

We used MBNL‐dependent cassette exons to generate adeno‐associated virus (AAV)‐compatible control elements (DMX^on^). Minigenes were tested in vitro using a Dox‐inducible MBNL1 cell model and induced pluripotent stem cell (iPSC)‐derived DM1 myotubes and in vivo using DM1 model mice following intramuscular and systemic AAV injection. DMX^on^ splicing, correction of endogenous splicing or skeletal muscle myotonia, and prevention of cardiac toxicity associated with therapeutic MBNL1 overexpression were assessed.

**Results:**

DMX^on^ cassettes respond to MBNL1 dose or expression of CUG repeat RNA. DMX^on^ controlled expression of therapeutic MBNL1 protein can improve skeletal muscle myotonia or prevent cardiac toxicity due to MBNL1 overexpression in mice.

**Interpretation:**

DMX^on^ control elements can increase the therapeutic window of viral‐based therapeutics in DM1, and activity is dependent upon delivered cargo and model severity. ANN NEUROL 2026;99:211–222

Genetically targeted therapies have become the standard‐of‐care for multiple inherited neuromuscular disorders, including spinal muscular atrophy,^1^ Duchenne muscular dystrophy,^2^ and transthyretin‐related amyloidosis neuropathy.^3^ Of these, viral‐based genetic therapies have the ability to deliver cargos throughout the body and provide prolonged expression when delivered to post‐mitotic cells. These advantages come with drawbacks, including immunogenicity that inhibits redosing regardless of efficacy, inability to remove or silence the therapy if toxic, and consequences of high vector exposure to multiple tissues where expression of the therapeutic gene may be unwarranted or unsafe.^4^ To improve efficacy and limit toxicity, efforts have focused on developing (i) novel viral capsids that reduce delivery to non‐target tissues,^5^, ^6^, ^7^, ^8^ (ii) promoters that direct tissue‐specific expression, or (iii) post‐transcriptional RNA regulatory elements that employ alternative splicing regulate gene expression.^9^, ^10^, ^11^, ^12^

Myotonic dystrophy type 1 (DM1) is an autosomal dominant genetic disorder that results in progressive muscular weakness and atrophy, cardiac conduction disease, and a multitude of other variable systemic effects.^13^ DM1 is caused by expansion of a CTG repeat in the 3′ untranslated region of *DMPK* that when transcribed accumulates as toxic CUG repeat RNAs. The mutant transcripts exert their toxicity through binding and sequestration of the muscleblind‐like (MBNL) family of splicing factor proteins, important players in pre‐mRNA processing.^14^, ^15^ Notably, the changes in alternative splicing that occur in DM1 due to MBNL loss‐of‐function have been shown to cause specific disease phenotypes^16^, ^17^ and serve as quantitative biomarkers of muscle weakness.^18^, ^19^

Genetic medicines to reduce toxic RNA accumulation are in clinical trials for DM1, and, similar to other inherited neuromuscular conditions, a long‐acting, viral‐based gene therapy may offer additional benefit. The application of gene therapy to DM1 poses several unique hurdles as DM1 is a multisystemic disease with high variability.^20^ Further, whereas MBNL supplementation is beneficial in skeletal muscle and the heart of DM1 models,^21^, ^22^ overexpression in wildtype mice has demonstrated toxicity^23^, ^24^ with cardiac tissue being particularly sensitive.^25^, ^26^ Thus, an MBNL replacement therapy will require regulated control of expression. To address this, we developed elements that post‐transcriptionally control a corrective genetic therapy for DM1 that responds to the amount of MBNL protein activity in situ (DMX^on^). We derived the DMX^on^ elements from known MBNL‐dependent alternative splicing events^18^, ^27^ and tested their activity using reporters or an MBNL therapeutic transgene. Here, we provide evidence that alternative splicing cassettes retain partial activity once removed from their natural context, that can be tuned to alter their responses in vitro and in vivo, and can be used to adjust protein output to limit toxicity or promote therapeutic efficacy in mouse models and induced pluripotent stem cell (iPSC)‐derived skeletal myotubes of DM1.

## Methods

### Plasmids and Adeno‐Associated Viral Vectors

Genomic sequence from *LDB3*, *MBNL1*, and *NFIX* were synthesized by gBlock using hg38 as a reference genome. Recombinant adeno‐associated virus (AAV) serotypes AAV2/9 or MyoAAV2A were generated at the CHOP Research Vector Core and resuspended in Diluent buffer. Vector titers were determined by droplet digital polymerase chain reaction (ddPCR) using primers targeting eGFP or MBNL1 transgenes. For MBNL1 overexpression vectors, sequence was amplified from Addgene plasmid #96906 (https://www.addgene.org/96906/)  ; a gift from Thomas Cooper.

### Induced Pluripotent Stem Cell Culture

The iPSC line NH50256 was obtained from the National Institute of Neurological Disorders and Stroke (NINDS) repository (stemcells.nindsgenetics.org). This line was generated from a patient with congenital myotonic dystrophy (CDM) with approximately 1700 CTG repeats. Prior to editing, the line underwent testing for Sendai viral vector clearance, mycoplasma, karyotyping, trilineage potential, and markers of stemness (SSEA and Tra) with the CHOP Stem Cell Core. To make isogenic control cells, Cas9‐mediated targeted insertion of a polyadenylation signal upstream of the expanded CTG repeat was performed, as previously described.^28^ Among the multiple clones generated, clone 11 was chosen for further study as it possessed a deletion of the expanded repeat as confirmed by long‐read sequencing. Lines were maintained in feeder‐free conditions with mTeSR1 on Matrigel and passaged by the clump method.

### Myoblast and Myotube Differentiation

Myoblasts/myotubes were generated by a combined inhibitor and PAX7 transgene overexpression method, as previously described.^29^ In brief, iPSCs were co‐transduced with FUGW‐rtTA and pSAM2‐iPAX7‐IRES‐tomato lentiviral vectors (a kind gift of Rita Perlingeiro, PhD). Embryoid bodies (EBs) were formed by incubating cells on orbital shaker with ROCK inhibitor (Y‐27632) for 2 days. EBs were then treated with GSK3β inhibitor (CHIR99021) in myogenic media for 2 days, then BMP (LDN193189) and TGFβ (SB431542) inhibitors were added. One day later, doxycycline was added to induce PAX7 expression for 2 additional days. EBs were then plated on gelatin‐coated plates and expanded in myogenic media supplemented with basic fibroblast growth factor (bFGF) for 4 days, and then sorted for tdTomato expression to enrich for myogenic progenitors. Progenitors were frozen prior to performing terminal differentiation for the experiments. For terminal differentiation, progenitors at passage 2+ were plated at 100‐ to 120,000 cells per well of a 24‐well plate and grown to high confluency for 3 days. Media was then changed to differentiation media and monitored for 5 to 10 days for formation of myotubes.

### Mouse Models and Viral Injections

All mice were bred and housed within an accredited Association for Assessment and Accreditation of Laboratory Animal Care (AAALAC) international facility. All procedures were approved by the CHOP Institutional Animal Care and Use Committee (IACUC) committee. Wild‐type animals were FVB/NJ strain and human skeletal actin with a long repeat (HSA^LR^) mice were the FVB/N‐Tg(HSA*LR)20bCath/J transgenic line (Jackson Laboratory). HSA^LR^ mice are transgenic for the human ACTA1 gene and proximal promoter with approximately 220 CTG repeats inserted into the 3′ untranslated region. The HSA^LR^ line was maintained as homozygous breeders, CTG repeat lengths were monitored in litters prior to selection of breeders, and periodic outcrossing back to FVB/NJ was performed.

Direct intramuscular (i.m.) injections were performed in tibialis anterior muscles in mice. In brief, mice were anesthetized with isoflurane, the distal hind limbs were shaved, and 5 × 10^9^ viral genomes (vgs) was brought to 20 μl in diluent buffer and was injected into the TA with a 30‐gauge insulin syringe.

Systemic delivery was performed by retro‐orbital injection. In brief, mice were anesthetized with isoflurane, 0.5% proparacaine anesthetic eye drops were delivered to the right eye and allowed to take effect for 2 to 5 minutes, a 30‐gauge insulin syringe was then passed into the retro‐orbital sinus, and 2 × 10^13^ vg/kg of viral vector in Diluent buffer to no more than 200 μl was injected.

### Immortalized Cell Culture

HEK293 cells were previously engineered to express tetracycline‐inducible HA‐MBNL1 protein.^30^ This line was used to test in vitro splicing and protein output of DMX^on^ minigenes. Cell cultures were maintained with DMEM (Gibco), 10% Tet‐System Approved FBS, 1% penicillin/streptomycin. For both RNA and protein experiments, 200,000 cells were plated in each well of a 24‐well plate. The following day, the cells were transitioned to doxycycline‐containing media and transfected 4 hours later with 250 ng plasmid/well using Lipofectamine 2000, per the manufacturer's protocol. Doxycycline‐containing media was replaced every 24 hours for experiments exceeding 24 hours.

### RNA Isolation and Reverse Transcription

For in vitro splicing assays in HA‐MBNL1 HEK293 cells, RNA was isolated 24 hours after plasmid transfection by Trizol, DNase treated using DNA‐free kit (Ambion), and 1 μg of RNA was reverse transcribed using a High‐Capacity cDNA Reverse Transcription kit (Applied Biosystems), per the manufacturer's protocol. Individual muscle or heart samples were homogenized using stainless steel beads in the TissueLyser LT (Qiagen) in Trizol, and then similarly treated with DNase, before 2 μg of RNA was reverse transcribed. The iPSC‐derived myotube RNA was isolated and DNase treated using the Zymo Quick RNA Miniprep kit. Reverse transcription used the High‐Capacity cDNA Reverse Transcription kit with variable input, depending on recovered concentration.

### Digital Droplet Polymerase Chain Reaction Analysis of Minigene Splicing

Polymerase chain reaction (PCR) primers were designed to amplify the region of the DMX^on^ switches (CK8_Mbnl_ddPCR_For: AGCGAATTAAACTCGAGACCA or CAG_ddPCR_For: TGT GCT GTC TCA TCA TTT TGG; Mbnl_ddPCR_Rev: GGGAAGTACAGCTTGAGGAAT; CK8_Nfix_ddPCR_For: AGCCAGCCAGCGAATTAAA, or For CAG_ddPCR_For: TGT GCT GTC TCA TCA TTT TGG; Nfix_ddPCR_Rev: GGAAGTGCAGGGCTGATG). HEX‐labeled probes were designed to bind to the alternate cassette exon (Mbnl_ddPCR_Cassette: /5HEX/AATCACTGA/ZEN/AGCCACCATGCTCGA/3IABkFQ/; NFIX_ddPCR_Cassette: /5HEX/AGTCAGGAA/ZEN/AGCTGGACTTCTGCA/3IABkFQ/), and FAM‐labeled probes to common sequence in flanking exons (Mbnl_ddPCR_Common: /56‐FAM/CAACCAGGC/ZEN/TGCAGC TGCA/3IABkFQ/; NFIX_ddPCR_Common: /56‐FAM/CGTCCACTT/ZEN/CGTTGGGCCAC/3IABkFQ/). All oligos were ordered from Integrated DNA Technologies. The cDNA samples were diluted up to 50 times to keep measurements within the linear quantification range, and mixed with Supermix (Bio‐Rad), primers, and probes. Droplets were read using the QX200 ddPCR system (Bio‐Rad).

### Semi‐Quantitative Real Time‐PCR of Alternative Splicing

PCR primers designed across mouse *Atp2a1* exon 21 to 23 (For: CTCATGGTCCTCAAGATCT CAC, Rev: GGGTCAGTGCCTCAGCTTTG), *Clcn1* exon 5 to 8 (For: TGAAGGAATACCTCACAC TCAAGG, Rev: CACGGAACACAAAGGCACTG), and *Clasp1* exon 19 to 21 (For: GCCAGTGCCA AATCCAAAG, Rev: GCTGAGACTGTGAAACCACT) were used to amplify cDNA generated from experimental animals using PrimeSTAR GXL DNA polymerase (Takara Bio). Products were visualized using 2.5% agarose gel electrophoresis stained with ethidium bromide. Band intensity was measured with Image J software.

### Quantitative RT‐PCR

Vector expression levels were measured using a CFX384 Real Time System (Bio‐Rad) using TaqMan Universal Master Mix II, no UNG (Applied Biosystems) with primer/probe set directed against eGFP (For: GAACCGCATCGAGCTGAA, Rev: TGCTTGTCGGCCATGATATAG, Probe: /56‐FAM/ATCGACTTC/ZEN/AAGGAGGACGGCAAC/3IABkFQ/), normalized to mouse β‐actin (Mm00607939_s1).

### Western Blot

Cell lysates were harvested by cell scraping in cold RIPA buffer (150 mM NaCl, 1% Igepal, 0.5% NaDOC, 0.1% SDS, 50 mM Tris pH 8.0) supplemented with 1X cOmplete protease inhibitors (Roche). Protein concentrations were measured using a Bio‐Rad RC DC assay and 20 μg of protein was then mixed with XT Sample Buffer (Bio‐Rad) and XT Reducing Agent (Bio‐Rad) and heated at 95°C for 5 minutes. SDS‐PAGE was then performed on Criterion XT 10% Bis‐Tris gels (Bio‐Rad), transfer to PVDF membrane was performed overnight at 4°C in Towbin buffer. Membranes were blocked with 5% non‐fat milk in Tris‐buffered saline‐Tween‐20 (TBST) before primary incubation with either 1:5000 anti‐MBNL1 (A2764; a kind gift from Charles Thornton, MD) or 1:5000 anti‐eGFP antibodies (Abcam, ab290), both diluted in block solution. HRP‐conjugated secondary antibodies were dilute in 5% milk and incubated at 1:10,000 for 1 hour at real time (RT) and exposed by Amersham ECL detection kit (Cytiva, RPN2105).

### Needle Electromyography

A Natus Nicolet EDX machine was used to perform needle electromyography (EMG) to assess myotonia in HSA^LR^ mice. Mice were anesthetized by isoflurane and maintained on a heating pad. Natus Teca Elite concentric needle electrode (length = 25 mm and diameter = 0.3 mm) was then inserted into the hind limb muscles to assess for myotonic discharges. Six total insertions were performed and video recorded per muscle. The percentage of insertions leading to a myotonic discharge was tallied. All measurements were done in a blinded fashion.

### Echocardiography

Mice were examined under isoflurane anesthesia, maintained on a warmed platform with electrocardiogram monitoring and heart rates above 400 bpm. M‐mode echocardiographic recordings were performed on short‐axis videos at the mid‐papillary level as well as short‐ and long‐axis 2D videos. These were used to measure left ventricular wall thickness and internal diameter, and then calculated the ejection fraction, cardiac output, and mass. All measurements and analysis of echocardiography were performed by a skilled and blinded echo technician.

### Statistical Analysis

All statistical analyses were performed using GraphPad Prism version 10. In experiments with more than 2 groups, analysis of variance (ANOVA) followed by Sidak's multiple comparisons testing was performed. Student's *t* test were used for all other pairwise testing. Comparison of survival was performed using log rank (Mantel‐Cox) testing. Further details are provided for individual experiments in the figure legends.

## Results

### Identification and In Vitro Evaluation of DMX^on^ Candidates

Sequestration of MBNL splicing factors on expanded CUG repeat RNA causes widespread mis‐splicing in the cells of patients with DM1. A subset of these mis‐splicing events involves inappropriate inclusion or exclusion of a cassette exon and each responds variably to changes in free MBNL concentration.^14^ Disease‐related changes in individual splice events are monitored by measuring the percent spliced in (PSI) or the percentage of transcripts containing the cassette exon. In this study, we sought to adapt MBNL‐dependent events as switches to increase therapeutic output when MBNL proteins were low and reduce output under an MBNL replete state.

To identify DMX^on^ candidates, we considered MBNL‐dependent alternative splicing events known to be biomarkers of disease severity in skeletal muscle.^17^, ^18^, ^27^, ^30^, ^31^, ^32^ These events correlate with weakness in the tibialis anterior (TA) muscles of patients with DM1^18^ and have been evaluated in MBNL loss‐of‐function and toxic RNA gain‐of‐function mouse models.^27^ For this work, we chose splicing events that: (1) are a single, relatively short alternate exon (ie, could not be a mutually exclusive or more complex event), (2) are excluded in the presence of MBNL protein, (3) have relatively large changes in percent spliced in (ΔPSI) across the spectrum of disease severity (>30% ΔPSI), and (4) are consistent across different skeletal muscles.^27^ Seven alternative splicing events fit these criteria; *LDB3* exon 8, *DCTN4* exon 6, *MPDZ* exon 27, *NFIX* exon 7, *JAG2* exon 10, *MAP3K4* exon 17, and *MBNL1* exon 5 (MBNL regulates its own activity through alternative splicing). Of these, we chose *LDB3* exon 8 because it had the largest ΔPSI, *MBNL1* exon 5 for its steep slope in response to changes in MBNL concentration and low EC50, (ie, it requires low levels of MBNL activity to achieve half‐maximal splicing activity^30^), and *NFIX* exon 7 for its high EC50 and relatively shallow slope of response to free MBNL levels (it requires high MBNL activity for maximal splicing). *JAG2* and *MAP3K4* had similar range and slope of response to *MBNL1* exon 5, albeit with larger alternate exons. *DCTN4* and *MPDZ* behaved similar to *NFIX* exon 7, but the small size of *DCTN4* exon 6 (21 nt) raised concern about the effects of modifications to the alternative exon, as sequences in alternate exons can alter splicing responses in MBNL‐dependent exclusion events.^14^

**FIGURE 1:**
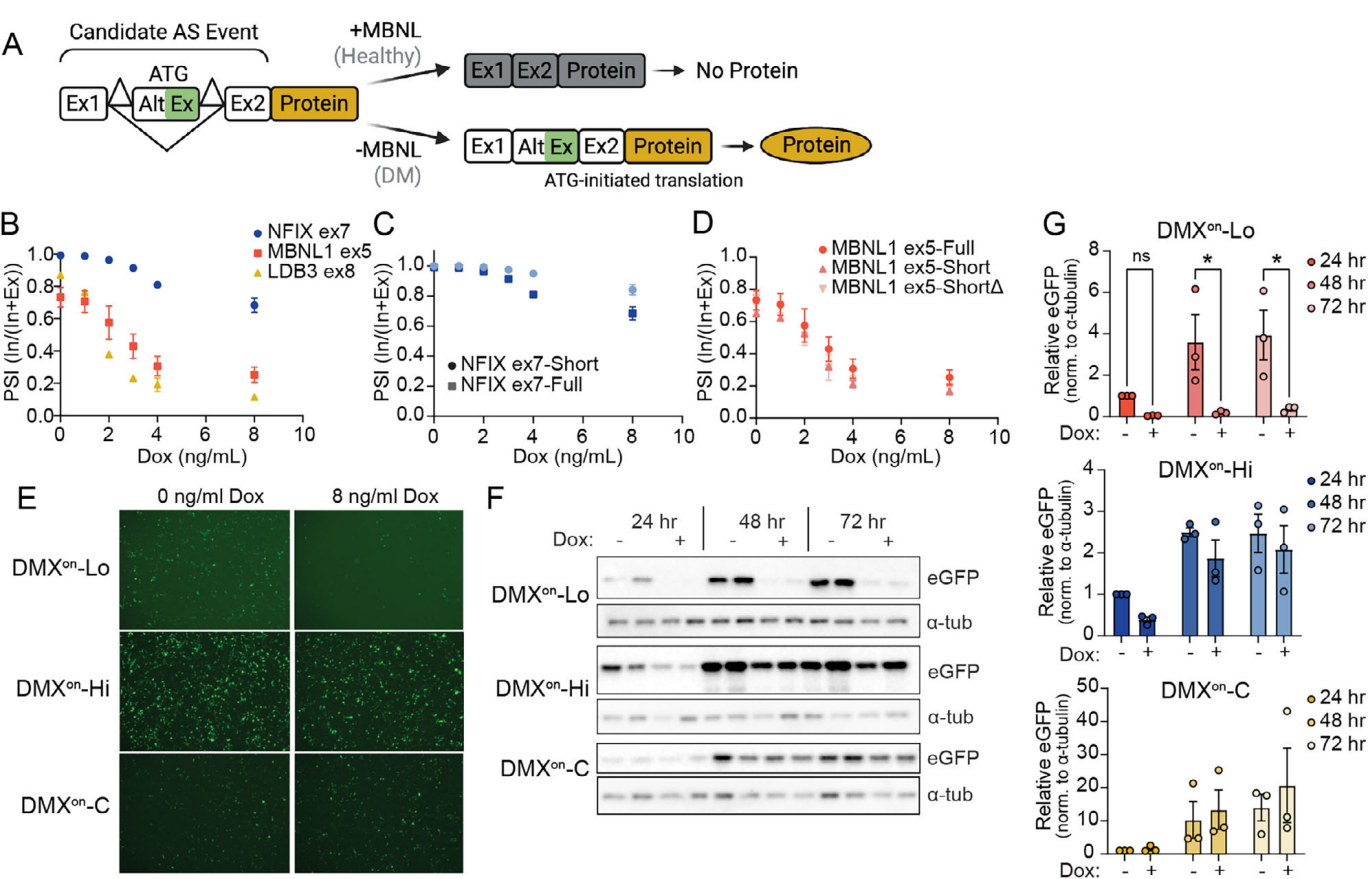
DMX^on^ design and in vitro testing. (A) Diagram of DMX^on^ splicing cassette design. Candidate MBNL‐dependent alternative splicing events were synthesized with the addition of a strong Kozak sequence inserted into the alternate exon (ATG). (B) Quantification of cassette exon inclusion measured as the PSI for full‐length DMX^on^ events (NFIX ex7, MBNL1 ex5, and LDB3 ex8) across a range of doxycycline doses in tet‐on HA‐MBNL1 HEK293 cells. (C) Comparison of NFIX ex7 dox dose–response between original (Full) and intron‐truncated (Short) variant. (D) Splicing dose response of MBNL1 ex5 variants with intronic truncations (Short) and mutation of 5′ splice site to remove start codon (ShortΔ) in exon 4. (E) Representative images of eGFP fluorescence from truncated variants at 24 hours following transfection in 0 ng/ml or 8 ng/ml doxycycline; DMX^on^‐Lo = MBNL1‐ShortΔ, DMX^on^‐C = MBNL1‐Short, DMX^on^‐Hi = NFIX‐Short. (F) Representative images of Western blots for eGFP protein across a 72‐hour time course. The α‐tubulin serves as loading control. (G) Quantification of eGFP Western blots, as shown in (F). Individual pairwise comparisons were performed with the Student's *t* test, *p < 0.05. DM = myotonic dystrophy; MBNL = muscleblind‐like; PSI = percent spliced in. [Color figure can be viewed at www.annalsofneurology.org]

We engineered minigenes from human gDNA sequence of *NFIX* exon 6 to 8, *MBNL1* exon 4 to 6, and *LDB3* exon 7 to 9 and modified them to include a strong Kozak sequence within the alternate exon to enable translation initiation only when the cassette exon is in frame with the reporter or therapeutic transgene coding sequence. First, the minigenes were cloned upstream of an eGFP reporter lacking a start codon (Fig 1A). A tetracycline‐inducible HA‐MBNL1 HEK293 cell line^30^ (a kind gift of Andrew Berglund) was used to test the responsiveness of the engineered cassettes to varying levels of MBNL (Supplementary Fig S1A). Similar to prior observations, the *NFIX* ex7 minigene had the narrowest dynamic range and highest EC50 of the 3 tested cassettes (Fig 1B). *LDB3* ex8 and MBNL1 ex5 showed broader dose responses to dox (PSI of LDB3 ex8 ranged from 87 to 11%, 0 to 8 ng/ml dox; MBNL1 ex5 ranged from 73 to 25%, 0 to 8 ng/ml dox) and significantly reduced inclusion in the presence of high levels of MBNL. Unexpectedly, gel analysis of *LDB3* ex8 splicing showed an extra, larger amplicon band suggesting downstream intron retention; therefore, this cassette was not evaluated further (see Supplementary Fig S1B).

MBNL‐dependent exon skipping events, such as those used in our cassettes, are modulated by MBNL binding within the alternative exon itself as well as the approximately 140 bp at the 3′ end of the upstream intron and approximately 100 bp at the 3’ end of the downstream intron.^14^, ^33^ Using this information and a map of putative MBNL binding sites in the engineered cassettes (https://rbpmap.technion.ac.il/), intronic regions were truncated. NFIX ex7‐short and MBNL1 ex5‐short showed modestly reduced ΔPSI across dox doses relative to the original versions (Fig 1C, D). We also generated MBNL1 ex5‐shortΔ wherein a methionine initiation codon flanking the 5′‐splice site at the boundary of exon/intron 4 was mutated. MBNL1 ex5‐ShortΔ retains the in vitro splicing response of MBNL1 ex5‐Short (see Fig 1D).

As MBNL1 ex5‐ShortΔ and NFIX ex7‐short respond to low or high concentrations of MBNL, respectively, we termed them DMX^on^‐Lo and DMX^on^‐Hi, and moved them forward for further study. As a control, we built a minigene retaining an upstream start codon for constitutive expression (DMX^on^‐C).

To assess if changing exon inclusion alters reporter protein production, fluorescent imaging of eGFP intensity at 24 hours and Western blot for eGFP protein across a 72‐hour time course were performed at no (0 ng/ml) or high (8 ng/ml) dox. Consistent with exon inclusion measurements, high dox concentrations suppressed eGFP production in DMX^on^‐Lo, but less so with DMX^on^‐Hi. As expected, DMX^on^‐C was not dox responsive (Fig 1E–G). These results demonstrate that disease‐related alternative splicing events can be isolated and adapted as switches to control protein expression in response to varying concentrations of free MBNL protein.

### In Vivo DMX^on^ Responds to Expanded Repeat RNA Accumulation and Shows Negative Feedback when Expressing Therapeutic MBNL1

To assess the ability of DMX^on^ to control transgene expression in vivo, we used a mouse model that expresses a transgene encoding a toxic CUG repeat embedded with human skeletal actin (human skeletal actin with a long repeat [HSA^LR^]). It is a skeletal muscle‐specific model of myotonic dystrophy and exhibits MBNL sequestration (low MBNL splicing activity levels), abnormal alternative splicing, and myotonia.^33^ Both DMX^on^‐Lo and DMX^on^‐Hi constructs were modified to include the CK8 promoter for muscle‐specific expression of the eGFP reporter or therapeutic MBNL1 protein. MBNL1 overexpression was chosen as a therapeutic strategy because it was previously shown to reduce muscle myotonia in HSA^LR^ mice following AAV delivery.^21^ DMX^on^ vectors were packaged into AAV9 and delivered by intramuscular injection at 5 × 10^9^ viral genomes into the TA muscles of wild type (WT) or HSA^LR^ mice.

**FIGURE 2:**
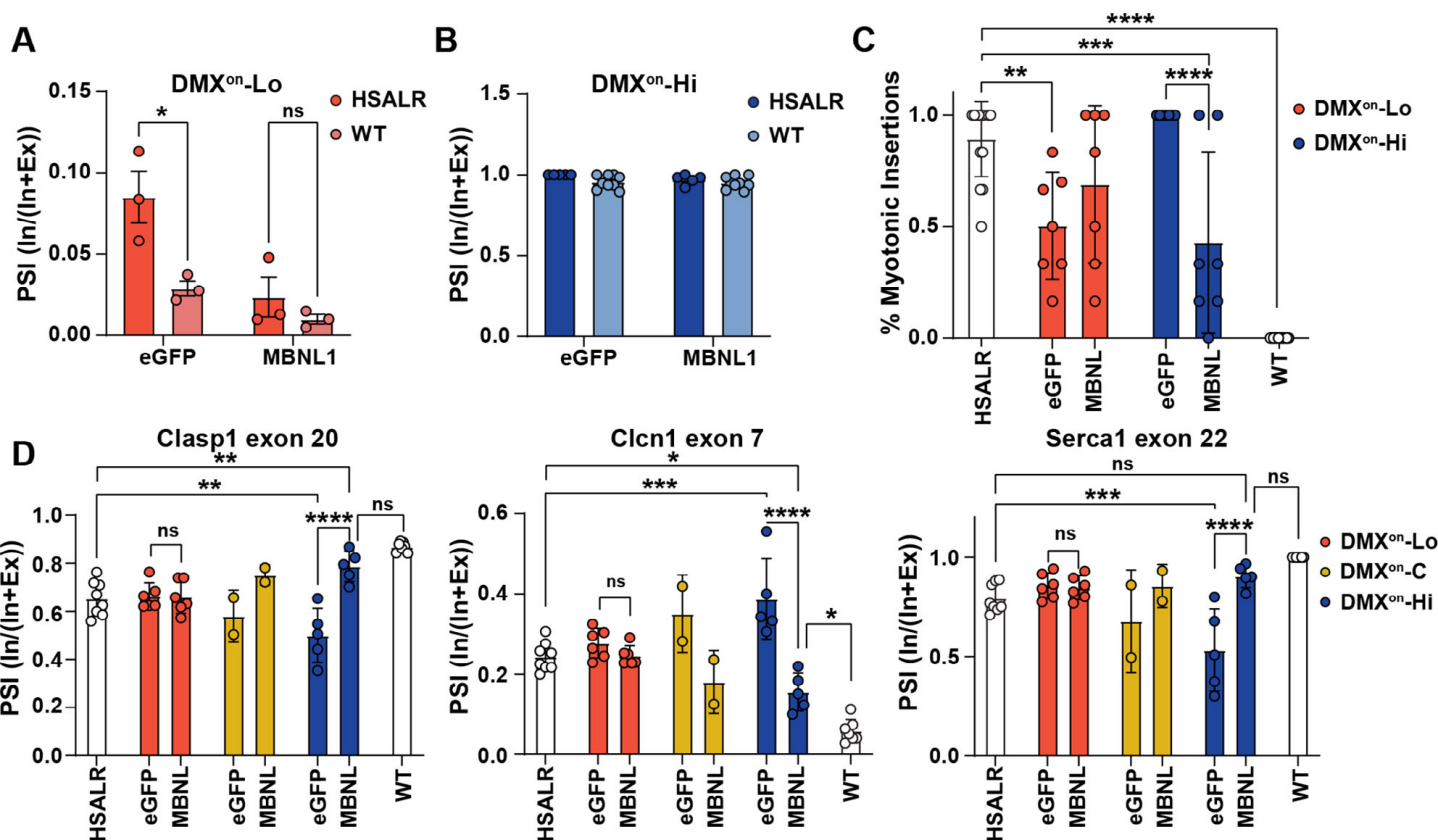
Intramuscular delivery of AAV9‐packaged DMX^on^ cassettes respond differently to expanded repeats in vivo. (A) Quantification of PSI of DMX^on^‐Lo (A) and DMX^on^‐Hi (B) by ddPCR in TA muscles of WT or HSA^LR^ mice when expressing eGFP reporter or MBNL1 therapeutic protein (n = 5‐6/group). (C) Quantification of myotonia by needle EMG as percentage of insertions leading to a myotonic discharge (n = 7–14/group). (D) Quantification of endogenous MBNL‐dependent splicing events (*Clasp1* exon 20, *Clcn1* exon 7, and *Atp2a1* exon 22) to assess therapeutic effect at 12 weeks post‐injection (n = 2–8). Individual pairwise comparisons were performed with the Student's *t* test for A and B. For C and D, one‐way ANOVA followed by Sidak's multiple comparisons was performed. *p < 0.05; **p < 0.01;***p < 0.005; ****p < 0.0001. ANOVA = analysis of variance; ddPCR = droplet digital polymerase chain reaction; EMG = electromyography; HSA^LR^ = human skeletal actin with a long repeat; MBNL = muscleblind‐like; PSI = percent spliced in; TA = tibialis anterior; WT = wild type. [Color figure can be viewed at www.annalsofneurology.org]

Initial evaluation of eGFP‐expressing vectors in TA muscles showed that the DMX^on^‐Lo.eGFP (switch.cargo) distinguished between WT and HSA^LR^ mice, with more cassette exon inclusion in HSA^LR^ mice, due to toxic RNA production and MBNL protein sequestration (Fig 2A). In addition, DMX^on^‐Lo.MBNL1 reduced cassette exon inclusion (see Fig 2A), suggesting sensitive feedback (lowering) on DMX^on^‐Lo splicing by AAV‐encoded MBNL1. In contrast, the DMX^on^‐Hi.eGFP cassette was less sensitive to differences in MBNL concentration, showing nearly 100% exon inclusion in both mouse strains. Further, DMX^on^‐Hi.MBNL1 was also insufficient to reduce exon inclusion (Fig 2B), suggesting constitutive expression of MBNL1 in AAV transduced tissues. These differences are consistent with observations of self‐inhibitory feedback observed in DMX^on^ constructs over a 72‐hour time course in vitro (Supplementary Fig S2).

The therapeutic impact of MBNL1 expression from the engineered switches was next assessed by measuring myotonia via needle EMG and evaluating known endogenous splicing events, *Clasp1* exon 20, *Clcn1* exon 7, and *Atp2a1* exon 22. At 12‐weeks post‐injection, a therapeutic effect was observed only with DMX^on^‐Hi.MBNL1 vectors (Fig 2C, D). Interestingly, DMX^on^‐Hi.eGFP treated animals had more severe myotonia and mis‐splicing of endogenous events, possibly due to binding of endogenous MBNL proteins to the DMX^on^‐Hi transcript. Mice treated with DMX^on^‐Lo.eGFP had a milder phenotype.

### Further Modification to DMX^on^ to Tune In Vivo Responsiveness

**FIGURE 3:**
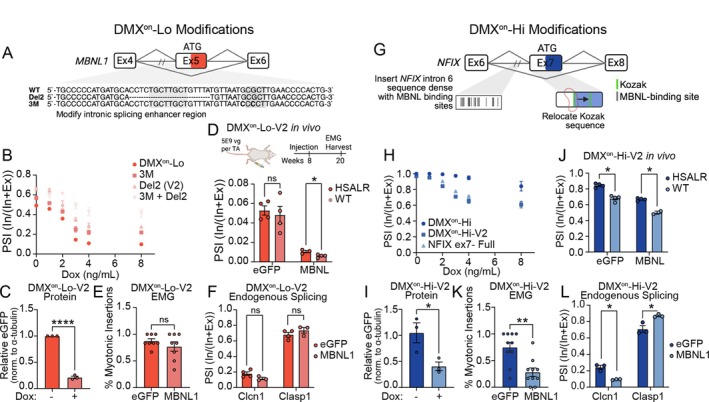
Changes to coding and non‐coding regions of DMX^on^ minigenes alter their response to MBNL concentration (A) Diagram of DMX^on^‐Lo minigene with Del2 and 3M mutations in intronic splicing silencer (adapted from Wagner et al 2016). (B) Quantification of exon inclusion of DMX^on^‐Lo mutation variants across a range of dox concentration (n = 3 per treatment/group) in HA‐MBNL1 HEK293 cells. Del2 is carried forward as DMX^on^‐Lo‐V2. (C) The eGFP protein output from DMX^on^‐Lo‐V2.eGFP minigene with 0 ng/ml or 8 ng/ml doxycycline after 72 hours (n = 3 per treatment) in HA‐MBNL1 HEK293 cells. (D) *Top* Experimental protocol. TA muscles of 8‐week‐old mice were injected with 5 × 10^9^ vg. EMG was performed at 12 weeks post‐injection, immediately prior to harvest. *Bottom* Splicing activity of CK8.DMX^on^‐Lo‐V2 cassettes at 12 weeks following i.m. injection in TA muscles of WT or HSA^LR^ mice (n = 4 muscles per treatment/genotype) shows no distinction between genotypes. (E) Myotonia measured by needle EMG 12 weeks post‐i.m. injection of DMX^on^‐Lo‐V2.eGFP or ‐.MBNL1 (n = 8 per treatment). (F) Quantification of endogenous MBNL‐dependent splicing events (*Clasp1* exon 20 and *Clcn1* exon 7) at 12 weeks following i.m. injection of DMX^on^‐Lo‐V2.eGFP or ‐.MBNL1 (n = 3/group). (G) Diagram of DMX^on^‐Hi minigene with alterations to the Kozak sequence location within exon 7 and addition of sequence in intron 6. (H) Quantification of DMX^on^‐Hi variant splicing, compared to original full‐length minigene, across a range of doxycycline concentrations (n = 3 per treatment/group). (I) Quantification of eGFP protein by Western blot of iCAG. DMX^on^‐Hi‐V2.eGFP minigene in HA‐MBNL1 HEK293 cells. (J) Measurement of exon inclusion of CK8. DMX^on^‐Hi‐V2 vectors following i.m. injection (n = 4 muscles per vector/genotype) shows suppression of inclusion by approximately 20%. Mice underwent the same experimental protocol outlined in D. (K) Myotonia measured by needle EMG 12 weeks post‐i.m. injection with DMX^on^‐Hi‐V2.eGFP or ‐.MBNL1 (n = 10 per treatment) showing suppression of myotonia in MBNL1 treated muscles. (L) Quantification of endogenous MBNL‐dependent splicing events at 12 weeks following i.m. injection with DMX^on^‐Hi‐V2.eGFP or ‐.MBNL1 (n = 3 / group) showing improvement toward WT levels. Individual pairwise comparisons were performed with the Student's *t* test, *p < 0.05, **p < 0.01, ****p < 0.0001. EMG = electromyography; HSA^LR^ = human skeletal actin with a long repeat; MBNL = muscleblind‐like; TA = tibialis anterior; vg = viral genome; WT = wild type. [Color figure can be viewed at www.annalsofneurology.org]

To adjust the responsivity of the DMX^on^‐Lo construct we mutated two MBNL‐binding motifs located in a known intronic splicing silencer region of intron 4 (Fig 3A), previously shown to decrease sensitivity to free MBNL1.^30^ Modifications included an 18 bp deletion (Del2) and introduction of 2 G>C mutations (3M). We also tested both alterations together (3M + Del2). All 3 modifications increased exon inclusion across a range of dox concentrations in the reporter cells (Fig 3B). However, the combination (3M + Del2) caused preference for an alternate splice acceptor (Supplementary Fig S3); therefore, this variant was not pursued further. Del2 alone retained the dynamic range of the original DMX^on^‐Lo construct as measured by ΔPSI and protein output in vitro (see Fig 3B, C) and was used as DMX^on^‐Lo‐version 2 (DMX^on^‐Lo‐V2) for in vivo testing.

Contrary to our in vitro testing results, DMX^on^‐Lo no longer distinguished between WT and HSA^LR^ TA muscles (Fig 3D). Further, DMX^on^‐Lo‐V2 did not significantly improve muscle myotonia (Fig 3E) or *Clasp1* exon 20 splicing and minimally altered endogenous *Clcn1* exon 7 splicing in both WT and HSA^LR^ mice (Fig 3F, Supplementary Fig S4A). As splicing is a co‐transcriptional event and subject to alterations in promoter activity, we examined if the responsiveness of DMX^on^‐Lo‐V2 in vivo may reflect use of a different promoter. We measured minigene splicing responses of the DMX^on^‐Lo‐V2 construct in vitro and observed identical splicing response using CK8 and the iCAG promoter (Supplementary Fig S4B).

Next, modifications to DMX^on^‐Hi were done. First, we moved the Kozak sequence in the alternative exon 7 to downstream of a putative MBNL1 binding site and re‐introduced approximately 1 kb of intron 6 sequence with a high density of putative MBNL1 binding sites (Fig 3G). These changes, named DMX^on^‐Hi‐V2, significantly reduced exon inclusion similar to the original full‐length hNFIX minigene (Fig 3H, I). Direct i.m. injection showed that DMX^on^‐Hi‐V2 now distinguished between WT and HSA^LR^ TA muscles (Fig 3J). In addition, treatment with DMX^on^‐Hi‐V2.MBNL1 vector showed a significant reduction in myotonic discharges and reversal of *Clasp1* exon 20 and *Clcn1* exon 7 mis‐splicing when compared with DMX^on^‐Hi‐V2.eGFP‐treated controls (Fig 3K, L).

### DMX^on^ ‐Controlled Systemic AAV Can Prevent MBNL1 Treatment‐Related Toxicity

**FIGURE 4:**
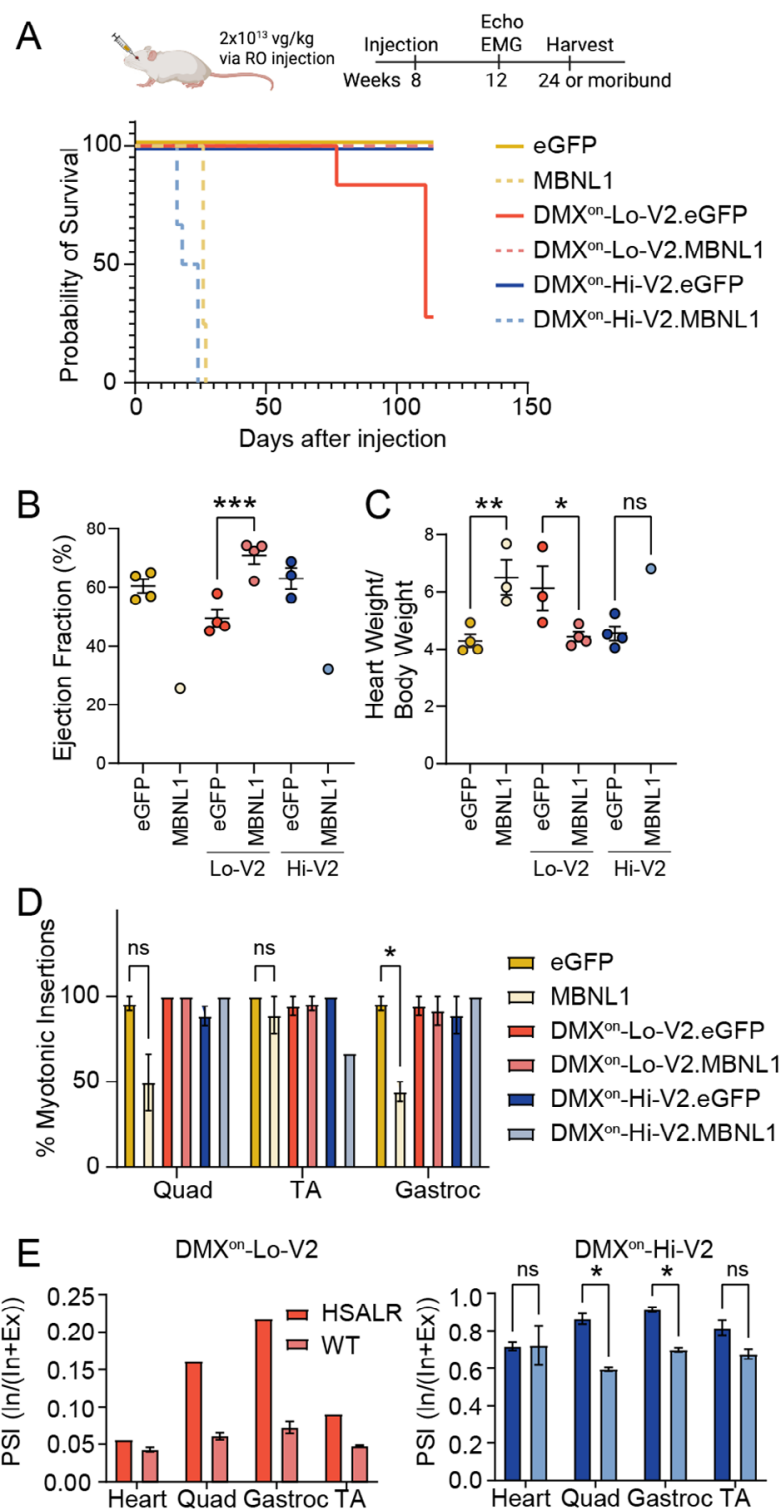
Mitigation of MBNL1 cardiac toxicity and DMX^on^ minigene responses following systemic delivery. (A) *Top* Experimental design. *Bottom* Survival curve following retro‐orbital injection of 2 × 10^13^ vg/kg at 8 weeks of age (switch.Cargo) (n = 4–6 per group). Animals were euthanized at 6 months of age. Log rank (Mantel‐Cox) testing showed significant differences between curves. (B) Quantification of ejection fraction by M‐mode cardiography 4 weeks post‐injection (n = 1–4 mice/group). (C) Quantification of heart weight/body weight following harvest (n = 4/group, except DMX^on^‐Hi‐V2.MBNL1 = 1 due to mortality). (D) Quantification of myotonia measured by needle EMG at 4 weeks post‐injection (n = 1–4 mice/group). (E) Quantification of minigene exon inclusion in the heart and 3 hindlimb muscles in DMX^on^‐Lo‐V2.eGFP (left) and DMX^on^‐Hi‐V2.eGFP (right) (n = 1–3/group) showing distinction between genotypes in skeletal muscles but not heart. Individual pairwise comparisons were performed with the Student's *t* test for E. For B through D, one‐way ANOVA followed by Sidak's multiple comparisons was performed. *p < 0.05; **p < 0.01;***p < 0.005. ANOVA = analysis of variance; EMG = electromyography; MBNL = muscleblind‐like; vg = viral genome. [Color figure can be viewed at www.annalsofneurology.org]

Viral‐mediated delivery of MBNL1 has previously been shown to improve the muscle and cardiac phenotypes in models of DM1^21^, ^22^, ^34^; however, others have reported severe cardiac toxicity of MBNL1 overexpression following systemic AAV‐mediated delivery in WT mice.^23^, ^25^, ^26^ To evaluate the ability of DMX^on^ to prevent a treatment‐related toxicity, we performed systemic delivery of DMX^on^.MBNL1 using myotropic AAV vectors (MyoAAV2A)^5^ via retro‐orbital injection in WT and HSA^LR^ mice (Fig 4A). As before, transgene expression was controlled by the CK8 promoter to drive strong expression in the heart and skeletal muscle. Importantly, the HSA^LR^ transgene is only expressed in skeletal muscle; therefore, DMX^on^ elements should respond similarly in the heart of WT and HSA^LR^ mice.

Consistent with prior reports in WT mice, we found that MBNL1 overexpression led to severe cardiac dysfunction with reduced left ventricular ejection fraction and death after 3 to 4 weeks (see Fig 4A, B). When DMX^on^.MBNL1 constructs were delivered, DMX^on^‐Lo‐V2.MBNL was protective while DMX^on^‐Hi‐V2.MBNL was not (see Fig 4A, B). Interestingly, DMX^on^‐Lo‐V2.eGFP also led to reduced survival after approximately 10 weeks post‐injection (see Fig 4A). Due to mortality, tissues were harvested early in the unregulated MBNL1 and DMX^on^‐Hi‐V2 groups at 22 to 24 days post‐injection, whereas the eGFP and DMX^on^‐Lo‐V2 groups were allowed to age for 101 to 114 days post‐injection before harvest. Despite the shortened time point, heart mass normalized to body weight demonstrated cardiomegaly in MBNL1 and DMX^on^‐Hi‐V2.MBNL1 mice (Fig 4C). Surprisingly, measurements in DMX^on^‐Lo‐V2.eGFP treated animals showed a moderate reduction of ejection fraction early on at 4 weeks post‐injection, and, albeit after considerably longer exposure to the vector, enlargement of heart size at harvest (see Fig 4B, C). Myotonia assessment in MBNL1 and DMX^on^‐Hi‐V2.MBNL1 treated groups was limited by small sample size or short treatment duration due to cardiac toxicity, but similar to results with i.m. injections above, systemic delivery of DMX^on^‐Lo‐V2.MBNL1 did not improve myotonia (Fig 4D).

Analysis of DMX^on^ splicing from the vector constructs was performed in heart, quadriceps, TA, and gastrocnemius muscles from animals treated with eGFP vectors. Both DMX^on^‐Lo‐V2 and DMX^on^‐Hi‐V2 splicing patterns were distinguishable between WT and HSA^LR^ skeletal muscles with a change in exon inclusion of 10 to 25%, depending on vector and specific hind limb muscle (Fig 4E). Interestingly, there was no significant difference in cassette splicing for either DMX^on^ in TA muscles between WT or HSA^LR^ mice, consistent with prior observations that HSA^LR^ transgene expression and disease severity is variable between muscles.^27^ As expected, DMX^on^ splicing in the heart of HSA^LR^ mice was similar to that observed in WT animals as they retain WT levels of MBNL activity. Vector expression of eGFP showed that the CK8 promoter drove ~8 times higher expression in the heart compared to skeletal muscles when normalized to β‐actin. Consistent with the use of a common promoter, expression levels among vectors were similar (Supplementary Fig S5).

### DMX^on^ Shows Stronger Response in Congenital Myotonic Dystrophy Myotubes

Our testing in HSA^LR^ mice showed that whereas DMX^on^ elements are responsive to toxic RNA accumulation, the magnitude of observed changes was limited and significantly less than that observed when modulating MBNL1 levels in vitro. This may reflect the fact that our DMX^on^ switches are derived from human sequence and may show reduced activity in mouse tissue, a common observation in non‐coding elements. Alternatively, HSA^LR^ mice are a relatively mild model of myotonic dystrophy, and DMX^on^ may show increased activity in a more severe model.

**FIGURE 5:**
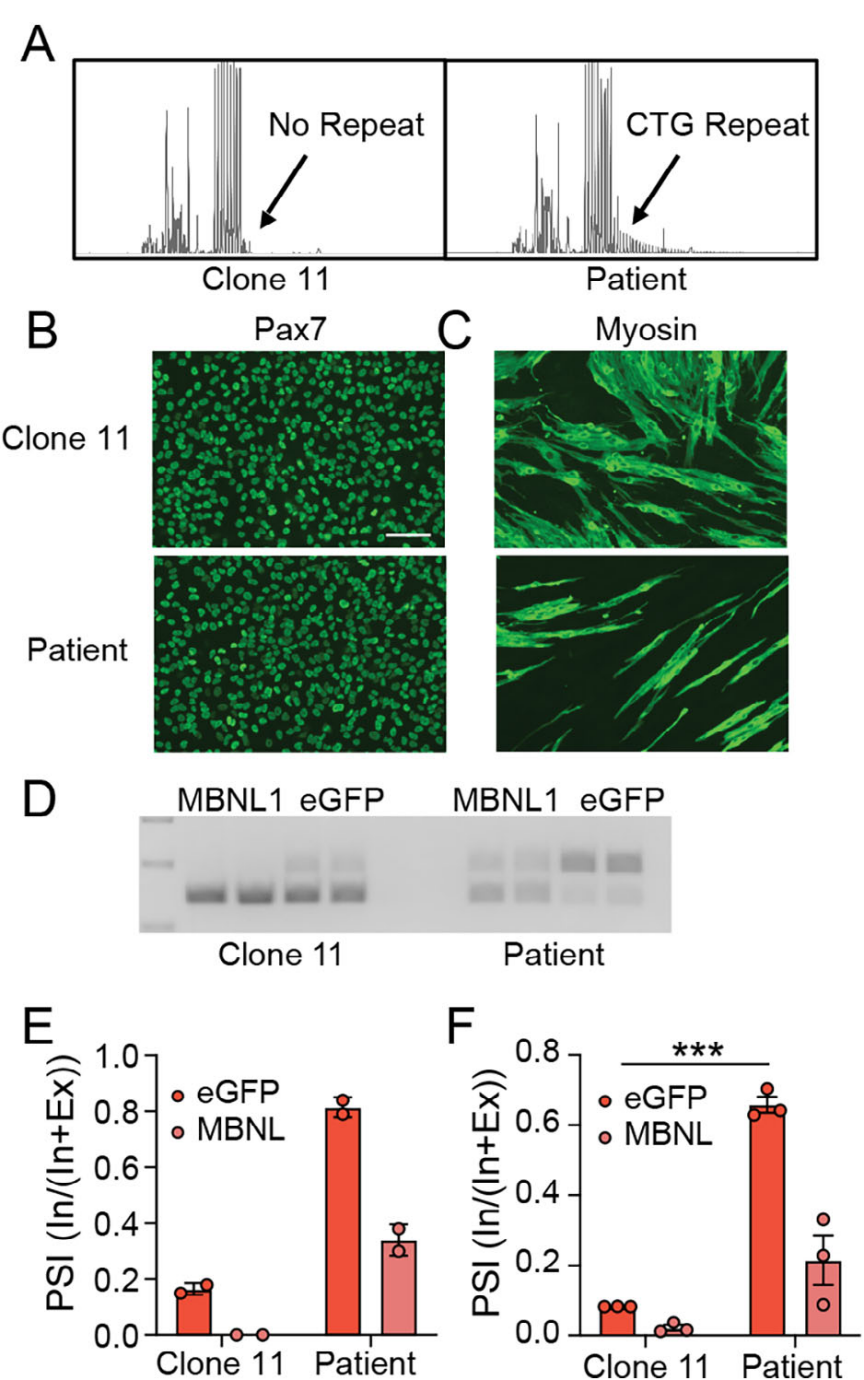
DMX^on^ response in congenital DM iPSC‐derived myotubes. (A) Repeat‐primed PCR demonstrating loss of repeat expansion in isogenic edited line, clone 11. Arrow points to loss of right tail segment that would indicate an expanded repeat. Representative images of (B) Pax7+ immunocytochemistry in myoblast cultures and (C) Myosin‐positive (MF‐20) multinucleated myotubes following 10 days of differentiation. (D) Representative image of RT‐PCR of DMX^on^‐Lo inclusion in myotubes when expressing either eGFP or therapeutic MBNL1. (E) Quantification of DMX^on^‐Lo exon inclusion in myotubes by gel intensities depicted in D. (F) Quantification of DMX^on^‐Lo inclusion in myotubes by ddPCR (n = 3 independent experiments). ***p < 0.005 with the Student's *t* test. iPSC = induced pluripotent stem cell; MBNL = muscleblind‐like; PCR = polymerase chain reaction; RT‐PCR = reverse transcription‐ polymerase chain reaction. [Color figure can be viewed at www.annalsofneurology.org]

To evaluate DMX^on^ response in a more severely affected human model system, we obtained an iPSC line derived from a patient with CDM (line NH50256, NINDS Cell Repository). CDM is the most severe form of myotonic dystrophy in which disease manifests within the first month of life and, similar to advanced adult disease, these infants show profound changes in MBNL‐dependent alternative splicing.^35^ Isogenic control lines were generated by Cas9‐mediated insertion of a polyadenylation signal upstream of the expanded CTG repeat in the DMPK 3′ untranslated region, as previously described.^36^, ^37^ This approach resulted in insertion of the polyadenylation signal with or without deletion of the expanded repeat. One of these lines, clone 11 had deletion of the expanded repeat DNA as determined by repeat primed PCR and Cas9‐targeted long read sequencing (Fig 5A, Supplementary Fig S6A) and loss of toxic RNA nuclear foci was verified by RNA fluorescence in situ hybridization (FISH; Supplementary Fig S6B).

Clone 11 and patient cells were programmed into myogenic precursor cells using tetracycline‐inducible *PAX7* overexpression^38^ before terminal differentiation into myotubes (Fig 5B). After 10 days in differentiation media, we evaluated the ability for both lines to form mature myotubes by probing with an antibody targeting myosin heavy chain (MF‐20; Fig 5C). Cultures at 10 days post‐differentiation were treated with 1 × 10^5^ vg/cell of DMX^on^‐Lo vectors. Vectors with transgenes expressed under control of the CK8 promoter were used to restrict expression to myotubes. Lines were incubated for an additional 7 days to allow for expression, and then the cells were harvested to evaluate the DMX^on^ cassette response. First, to confirm the size of spliced DMX^on^ products and rule out viral gDNA contamination, we performed reverse transcription‐PCR (RT‐PCR). This showed expected amplicon sizes and a clear differentiation of isogenic control cells from patient myotubes (Fig 5D, E). We then performed ddPCR to more accurately quantify exon inclusion (Fig 5F). The DMX^on^‐Lo switch showed strong discrimination between the patient's and the isogenic control cells with inclusion of the cassette exon in 64% of transcripts in the patient cells compared with only 8% in the healthy controls. Transgene‐derived MBNL1 protein expression reduced cassette exon inclusion to 23% in patient cells and 1% in the healthy controls, supporting negative feedback of the DMX^on^ cassette in human cells (see Fig 5F).

## Discussion

Viral‐based gene therapy has shown clinical efficacy for several fatal genetic neuromuscular diseases.^1^, ^39^ As with all therapeutics, application requires finding an appropriate dose to drive adequate transgene expression while limiting related toxicity.^39^, ^40^ Dose identification in myotonic dystrophy is complicated by high clinical variability correlated to changes in MBNL‐dependent RNA processing.^18^, ^19^, ^27^ Here, we took advantage of disease‐related alternative splicing changes to generate DMX^on^, a regulatory switch that tunes transgene output in response to MBNL levels, a proxy for disease severity. Using cell and animal model systems, we show that DMX^on^ switches respond to changes in MBNL1 concentration and accumulation of expanded repeat RNA.

Incorporation of splice cassettes to control spatial or temporal transgene expression has been previously reported using both drug‐ and cell‐specific approaches.^9^, ^10^, ^11^, ^41^, ^42^, ^43^ While our work was underway, a similar strategy for myotonic dystrophy was published by Rogalska et al.^12^ Different from our design, the authors incorporated a stop codon into the alternate exon, where exon inclusion leads to early termination generating a small, non‐functional peptide in WT cells. DMX^on^ expands on our previously published X^on^ system,^9^ where exon exclusion is predicted to prevent peptide production.

In this study, our initial variants and downstream modifications were informed using published work.^14^, ^18^, ^27^, ^30^ Testing of isolated DMX^on^ cassettes in vitro demonstrated strong MBNL dose‐dependent splicing, with a magnitude of exon response of 30 to 50% (see Figs 1, 3). Surprisingly, this response was reduced in vivo to approximately 5 to 15% in TA muscles and maximally 27% in quadriceps (see Figs 2, 3, 4) in HSA^LR^ mice. Consistent with our observations in human DM1 cells (see Fig 5), one possible explanation is the relatively mild disease in this model,^27^ especially in TA muscle.^44^ Based on these findings, further optimization of DMX^on^ may benefit from testing in a more severe model of DM1, such as *Mbnl1* knockout, and by high‐throughput mutagenesis to identify more active cassettes in vivo.^11^, ^41^

To test the therapeutic potential of DMX^on^ we used MBNL1 overexpression for 2 reasons: (1) its ability to reverse skeletal muscle myotonia in the HSA^LR^ mouse model^21^ and (2) its ability to cause cardiac toxicity with systemic AAV delivery in WT mice.^23^, ^25^, ^26^ Although DMX^on^‐Hi could retain MBNL1 output to treat skeletal muscle myotonia and RNA mis‐splicing, heart toxicity was still observed likely due to expression of MBNL1 over WT levels in the HSA^LR^ heart. To the contrary, DMX^on^‐Lo could reduce the output of MBNL1 to prevent cardiac toxicity but did not correct muscle myotonia. Thus, an individual DMX^on^ did not achieve both goals in the HSA^LR^ model. Importantly, this experiment was designed to examine the reactivity of DMX^on^, and interpretation of the observed cardiac toxicity is limited, as MBNL1 overexpression has been shown to be beneficial in cardiac models of DM1.^22^

Taken together, our data support that DMX^on^ can respond to and tune the output of a viral‐based gene therapy dependent on disease‐severity in DM1. Further modifications of DMX^on^ may provide a more comprehensive and efficacious treatment strategy for DM1, a highly variable multisystemic disease.

## Author Contributions

S.T.C., E.M.C, and B.L.D. contributed to the conception and design of the study. S.T.C., E.M.C, R.C.G. acquired and analyzed the data. S.T.C., E.M.C, and B.L.D. drafted the manuscript and figures. [Correction added on 14 January 2026, after first online publication: Author contribution text has been revised in this version.]

## Potential Conflicts of Interest

The authors report no conflicts of interested related to this work.

## Supporting information


**Supplementary Figure S1.** Doxycycline‐inducible MBNL1 HEK293 cells and *LDB3* abnormal splicing. (A) Representative Western blot of Mbnl1 protein expression in tetracycline‐inducible HA‐MBNL1 HEK 293 cells across a range of doxycycline concentrations. (B) Representative agarose gel of RT‐PCR to measure alternative splicing of *LDB3* exon 8, arrow demarcates a larger, unpredicted product.
**Supplementary Figure S2.** DMX^on^ splicing regulation by cell‐ or transgene‐derived MBNL1 overexpression. Representative RT‐PCR agarose gels of (A) DMX^on^‐Lo, (B) DMX^on^‐C, and (C) DMX^on^‐Hi splicing over a 72‐hour time course in response to cell (dox induced) or transgene‐derived MBNL1 protein in HA‐MBNL1 tet‐inducible cells.
**Supplementary Figure S3.** Aberrant use of upstream alternative splice site in combined Del2 and 3M variant. (A) Representative RT‐PCR gel of DMX^on^‐Lo Del2 and 3M + Del2 across a range of doxycycline. (B) Same as (A), but without doxycycline in order to isolate the larger amplicon for sequencing. (C) Sequence of intron 6 – exon 7 junction with Sanger sequencing below showing inclusion of upstream sequence and strong Kozak sequence and start codon (red box).
**Supplementary Figure S4.** DMX^on^‐Lo‐V2 minor impact of endogenous splicing in WT mice and reporter splicing is not reliant on promoter. (A) Quantification of endogenous splicing of mouse *Clcn1* exon 7 and *Clasp1* exon 20 in eGFP or MBNL1 treated TA muscles in WT mice (n = 4/group). (B) Quantification of exon inclusion by ddPCR across a range of doxycycline in tetracycline‐inducible HA‐MBNL1 HEK 293 cells when driven by either iCAG or CK8 promoter.
**Supplementary Figure S5.** eGFP RNA expression is similar between vectors when delivered systemically. Quantification of eGFP expression in various tissues following systemic delivery of constitutive or DMX^on^ regulated vectors in WT (denoted) or HSA^LR^ mice.
**Supplementary Figure S6.** Insertion of polyadenylation signal by Cas9‐mediated targeting leads to CTG repeat deletion and loss of RNA foci in CDM iPSCs. (A) Coverage maps of long‐read sequencing of DMPK repeat region in congenital DM1 patient iPSC (above) and edited clone 11 (below). (B) Representative images of RNA fluorescence in situ hybridization using a Cy3‐conjugated (CAG) 5 probe (PNA Bio) in CDM iPSC or edited clone 11 cells.

## Data Availability

Data will be made available upon publication according to CHOP policies.
