# Supplementary material for: Co‐Opting MBNL‐Dependent Alternative Splicing Cassette Exons to Control Gene Therapy in Myotonic Dystrophy
Source: Ann Neurol. 2025 Aug 29;99(1):211–22. doi: 10.1002/ana.78024 (PMC12946605; doi:10.1002/ana.78024)
Supplement: Supplementary file 1 — Supplementary Figure S1. Doxycycline‐inducible MBNL1 HEK293 cells and LDB3 abnormal splicing. (A) Representative Western blot of Mbnl1 protein expression in tetracycline‐inducible HA‐MBNL1 HEK 293 cells across a range of doxycycline concentrations. (B) Representative agarose gel of RT‐PCR to measure alternative splicing of LDB3 exon 8, arrow demarcates a larger, unpredicted product. Supplementary Figure S2. DMXon splicing regulation by cell‐ or transgene‐derived MBNL1 overexpression. Representative RT‐PCR agarose gels of (A) DMXon‐Lo, (B) DMXon‐C, and (C) DMXon‐Hi splicing over a 72‐hour time course in response to cell (dox induced) or transgene‐derived MBNL1 protein in HA‐MBNL1 tet‐inducible cells. Supplementary Figure S3. Aberrant use of upstream alternative splice site in combined Del2 and 3M variant. (A) Representative RT‐PCR gel of DMXon‐Lo Del2 and 3M + Del2 across a range of doxycycline. (B) Same as (A), but without doxycycline in order to isolate the larger amplicon for sequencing. (C) Sequence of intron 6 – exon 7 junction with Sanger sequencing below showing inclusion of upstream sequence and strong Kozak sequence and start codon (red box). Supplementary Figure S4. DMXon‐Lo‐V2 minor impact of endogenous splicing in WT mice and reporter splicing is not reliant on promoter. (A) Quantification of endogenous splicing of mouse Clcn1 exon 7 and Clasp1 exon 20 in eGFP or MBNL1 treated TA muscles in WT mice (n = 4/group). (B) Quantification of exon inclusion by ddPCR across a range of doxycycline in tetracycline‐inducible HA‐MBNL1 HEK 293 cells when driven by either iCAG or CK8 promoter. Supplementary Figure S5. eGFP RNA expression is similar between vectors when delivered systemically. Quantification of eGFP expression in various tissues following systemic delivery of constitutive or DMXon regulated vectors in WT (denoted) or HSALR mice. Supplementary Figure S6. Insertion of polyadenylation signal by Cas9‐mediated targeting leads to CTG repeat deletion and loss o [file ANA-99-211-s001.docx]

**Supplemental Figures**

**Co-opting MBNL-dependent alternative splicing cassette exons to control gene therapy in myotonic dystrophy**

Carrell ST^*1,2^, Carrell EM^*2^, Giovenco, R^2^, Davidson BL^2,3,&^

^1^Department of Neurology, University of Pennsylvania, Philadelphia, PA, 19104, United States

^2^ Raymond G. Perelman Center for Cellular and Molecular Therapeutics, The Children’s Hospital of Philadelphia, Philadelphia, PA 19104, United States.

^3^Department of Pathology and Laboratory Medicine, University of Pennsylvania, Philadelphia, PA 19104, United States

**
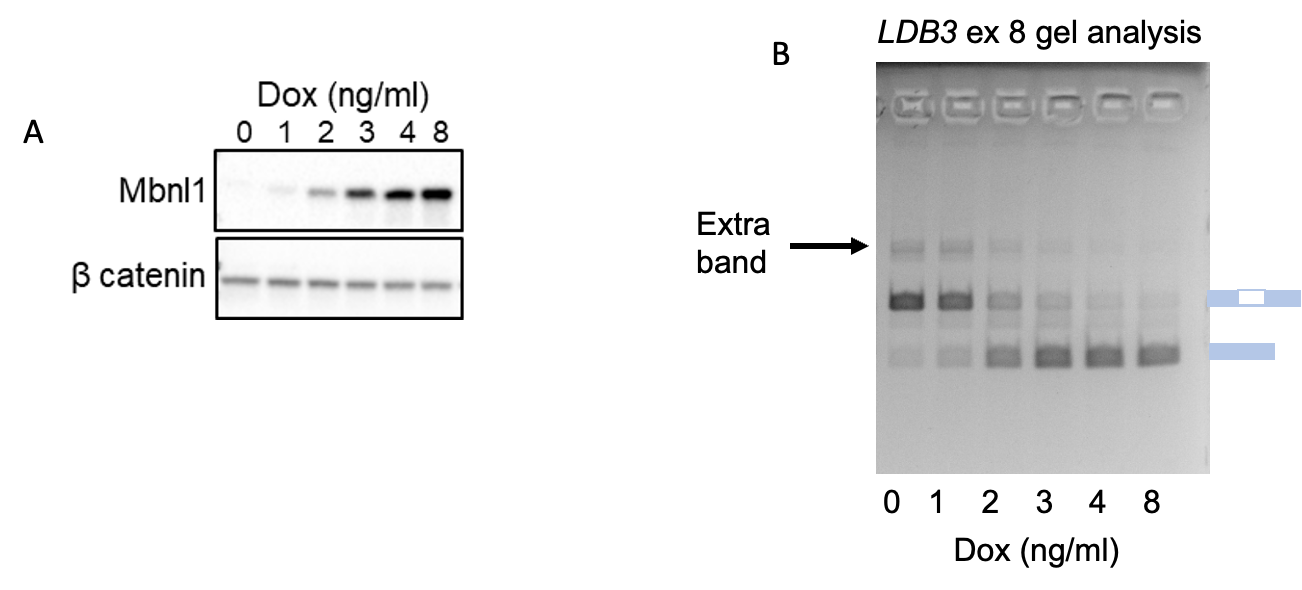
**

**Supplemental Figure 1. Doxycycline-inducible MBNL1 HEK293 cells and *LDB3* abnormal splicing.** (A) Representative Western blot of Mbnl1 protein expression in tetracycline-inducible HA-MBNL1 HEK 293 cells across a range of doxycycline concentrations. (B) Representative agarose gel of RT-PCR to measure alternative splicing of *LDB3* exon 8, arrow demarcated larger product.


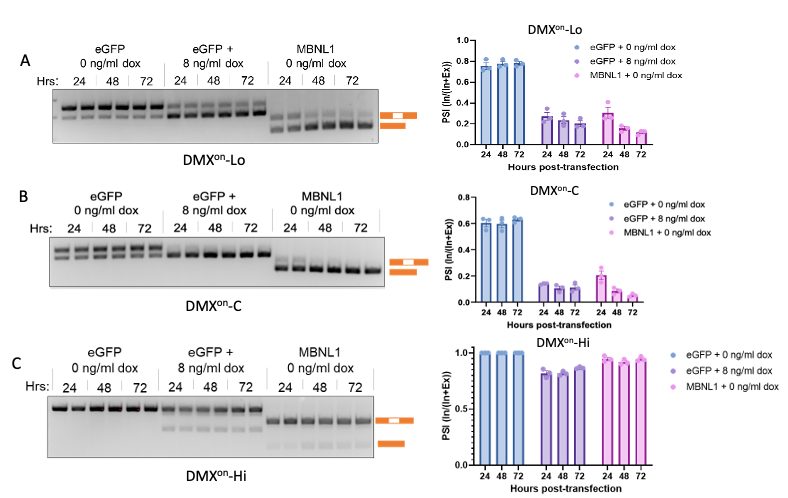


**Supplemental Figure 2.** **DMX^on^ self-regulation to MBNL1 overexpression.** Representative RT-PCR agarose gels of (A) DMX^on^-Lo, (B) DMX^on^-C, and (C) DMX^on^-Hi splicing over a 72-hour time course in response to MBNL1 protein driven in HA-MBNL1 tet-inducible cells or from self-expression of MBNL1.


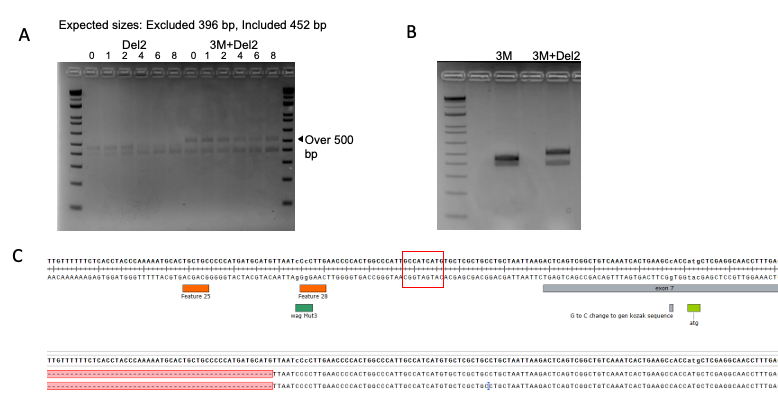


**Supplemental Figure 3. Aberrant use of upstream alternative splice site in combined Del2 and 3M variant.** (A) Representative RT-PCR gel of DMX^on^-Lo Del2 and 3M+Del2 across a range of doxycycline. (B) Same as (A), but without doxycycline in order to isolate the larger amplicon for sequencing. (C) Sequence of intron 6 – exon 7 junction with Sanger sequencing below showing inclusion of upstream sequence and strong Kozak sequence and start codon (red box).


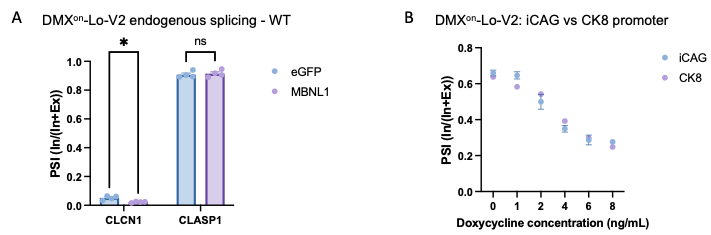


**Supplemental Figure 4. DMX^on^-Lo-V2 minor impact of endogenous splicing in WT mice and reporter splicing is not reliant on promoter.** (A) Quantification of endogenous splicing of mouse *Clcn1* exon 7 and *Clasp1* exon 20 in eGFP or MBNL1 treated TA muscles in WT mice (n = 4 / group). (B) Quantification of exon inclusion by ddPCR across a range of doxycycline in tetracycline-inducible HA-MBNL1 HEK 293 cells when driven by either iCAG or CK8 promoter.


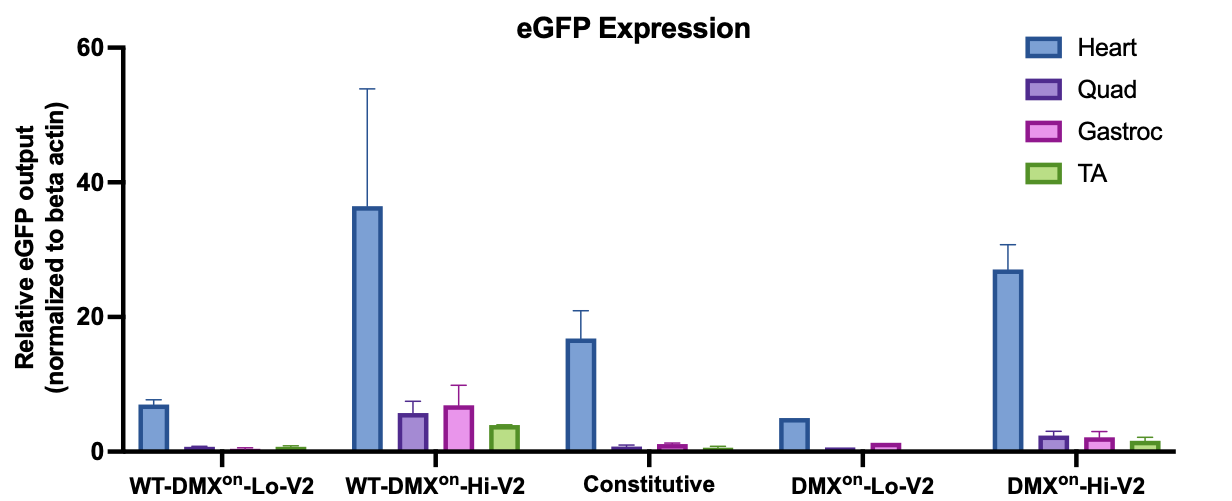


**Supplemental Figure 5. eGFP expression is similar between vectors when delivered systemically.** Quantification of eGFP expression in various tissues following systemic delivery of constitutive or DMX^on^ regulated vectors in WT (denoted) or HSALR mice.

**
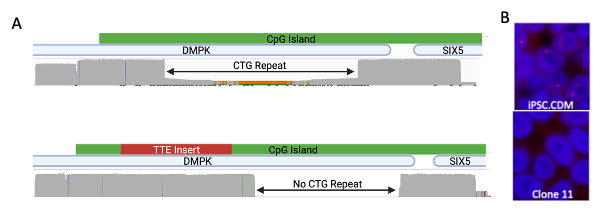
**

**Supplemental Figure 6. Insertion of polyadenylation signal by Cas9-mediated targeting leads to CTG repeat deletion and loss of RNA foci in CDM iPSCs.** (A) Coverage maps of long-read sequencing of DMPK repeat region in congenital DM1 patient iPSC (above) and edited clone 11 (below). (B) Representative images of RNA fluorescence in situ hybridization using a Cy3-conjugated (CAG)5 probe (PNA Bio) in CDM iPSC or edited clone 11 cells.
